# Aryl Hydrocarbon Receptor Interacting Protein Maintains Germinal Center B Cells through Suppression of BCL6 Degradation

**DOI:** 10.1016/j.celrep.2019.04.014

**Published:** 2019-04-30

**Authors:** Dijue Sun, Urszula Stopka-Farooqui, Sayka Barry, Ezra Aksoy, Gregory Parsonage, Anna Vossenkämper, Melania Capasso, Xinyu Wan, Sherine Norris, Jennifer L. Marshall, Andrew Clear, John Gribben, Thomas T. MacDonald, Christopher D. Buckley, Márta Korbonits, Oliver Haworth

**Affiliations:** 1 Center of Biochemical Pharmacology, William Harvey Research Institute, Barts and the London School of Medicine and Dentistry, Queen Mary University of London, London EC1M 6BQ, UK; 2 Endocrinology, William Harvey Research Institute, Barts and the London School of Medicine and Dentistry, Queen Mary University of London, London EC1M 6BQ, UK; 3 Experimental Medicine & Rheumatology, William Harvey Research Institute, Barts and the London School of Medicine and Dentistry, Queen Mary University of London, London EC1M 6BQ, UK; 4 Center for Immunobiology, Blizard Institute, Barts and the London School of Medicine and Dentistry, Queen Mary University of London, London EC1M 6BQ, UK; 5 Barts Cancer Institute, Barts and the London School of Medicine and Dentistry, Queen Mary University of London, London EC1M 6BQ, UK; 6 Institute of Inflammation and Ageing, University of Birmingham, Birmingham B15 2TT, UK; 7 Department of Biological Sciences, Westminster University, London W1W 6UW, UK

**Keywords:** AIP, BCL6, FBXO11, UCHL1, ubiquitination, lymphoma

## Abstract

B cell lymphoma-6 (BCL6) is highly expressed in germinal center B cells, but how its expression is maintained is still not completely clear. Aryl hydrocarbon receptor interacting protein (AIP) is a co-chaperone of heat shock protein 90. Deletion of *Aip* in B cells decreased BCL6 expression, reducing germinal center B cells and diminishing adaptive immune responses. AIP was required for optimal AKT signaling in response to B cell receptor stimulation, and AIP protected BCL6 from ubiquitin-mediated proteasomal degradation by the E3-ubiquitin ligase FBXO11 by binding to the deubiquitinase UCHL1, thus helping to maintain the expression of BCL6. AIP was highly expressed in primary diffuse large B cell lymphomas compared to healthy tissue and other tumors. Our findings describe AIP as a positive regulator of BCL6 expression with implications for the pathobiology of diffuse large B cell lymphoma.

## Highlights

• AIP supports BCL6 expression in germinal center and DLBCL cells
• AIP inhibits BCL6 degradation by regulating the deubiquitinase UCHL1
• AIP is highly expressed in DLBCL and is associated with decreased survival in DLBCL

## Graphical Abstract

**Figure:**
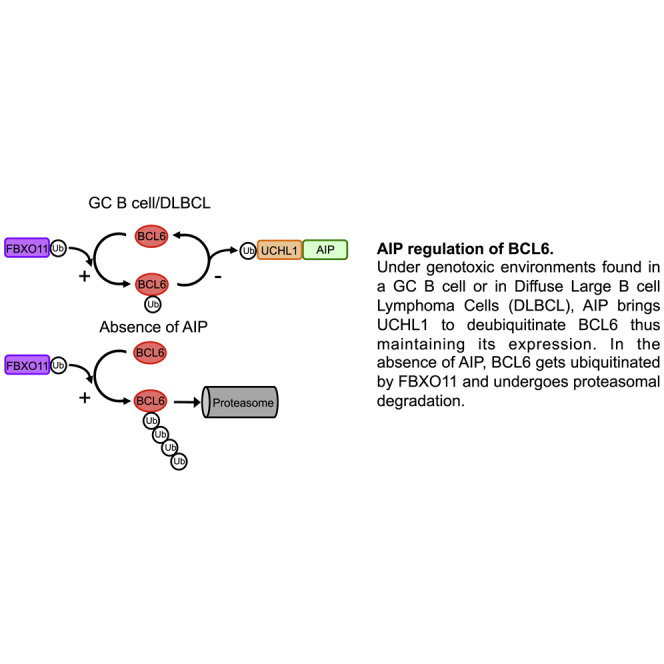

## Teaser

BCL6 overexpression contributes to the pathobiology of diffuse large B cell lymphoma (DLBCL). Sun et al. find that the co-chaperone aryl hydrocarbon receptor interacting protein (AIP), whose high expression is associated with reduced survival of DLBCL patients, helps maintain BCL6 expression by facilitating the removal of ubiquitin from BCL6.

## Introduction

Chaperone molecules play a crucial role in cellular homeostasis, stabilizing labile proteins during periods of cellular stress. Heat shock protein 90 (HSP90) is central to the maintenance of cellular homeostasis (Taipale et al., 2010), and studies have indicated that HSP90 can bind to 60% of the human kinome and 30% of E3 ubiquitin ligases (Schopf et al., 2017, Taipale et al., 2014). Co-chaperone proteins assist chaperone molecules in their supply and binding of specific client proteins to chaperone molecules (Trepel et al., 2010), yet the precise molecular function by which co-chaperones of HSP90 operate is still poorly understood (Schopf et al., 2017).

Aryl hydrocarbon receptor (AHR) interacting protein (AIP) is a conserved co-chaperone protein that binds to many proteins, including AHR and HSP90 (Stockinger et al., 2014, Trivellin and Korbonits, 2011). Individuals carrying monoallelic loss-of-function mutations in *AIP* (*AIP* carriers) are predisposed to young-onset, aggressive, usually growth-hormone-secreting pituitary adenomas that often result in acromegalic gigantism (Caimari and Korbonits, 2016, Vierimaa et al., 2006). Our group set out to understand the function of AIP in regulating adaptive immune responses.

Because of the well-described role of AHR regulating T helper 17 (T_H_17) cells (Li et al., 2011, Veldhoen et al., 2008), we started to examine the function of AIP on T cells and used the *Rag1*^*Cre/+*^ mouse strain to study its function in T cells. Unexpectedly, however, we noticed that deletion of *Aip* had an effect on B cells, and we sought to investigate this function in more detail. Germinal centers (GCs) are structures within secondary lymphoid tissues that are vital for the development of effective adaptive immune responses against pathogens (Allen et al., 2007, Victora and Nussenzweig, 2012). GCs are challenging environments for lymphocytes. B cells, upon activation, enter GCs where they undergo rapid proliferation, class switch recombination, somatic hyper-mutation, and affinity maturation, all of which place considerable genotoxic stress on B cells (Allen et al., 2007, Victora and Nussenzweig, 2012). Inhibitors of HSP90 have been shown to be effective in inducing apoptosis of B cell lymphomas that have a GC origin and overexpress B cell lymphoma-6 (BCL6) protein (Cerchietti et al., 2009).

BCL6 is a master regulator of GC B cell phenotype (Bunting et al., 2016, Dent et al., 1997, Ye et al., 1997). By repressing transcription of pro-apoptotic genes such as *TP53* (Basso and Dalla-Favera, 2015), BCL6 enables GC B cells to tolerate genotoxic stress as they undergo rapid proliferation with somatic hyper-mutation and class switch recombination (Basso and Dalla-Favera, 2015). Accordingly, BCL6 upregulation is commonly found in B cell lymphomas of GC origin (Baron et al., 1993, Basso and Dalla-Favera, 2015).

Here, we deleted *Aip* in mouse B cells, which led to suboptimal adaptive immune responses, via altered AKT signaling and by controlling the expression of BCL6 in GC B cells. We show that AIP protects BCL6 from E3 ubiquitin ligase FBXO11-induced proteasomal degradation via binding the deubiquitinase UCHL1. Together, these results demonstrate AIP as a positive regulator of BCL6.

## Results

### AIP Regulates Adaptive Immune Responses

To assess the impact of AIP on adaptive immune responses, we crossed *Aip*^*fl/fl*^ mice with *Rag1*^*Cre/+*^ mice generating mice carrying a conditional homozygous deletion of *Aip* in T and B cells (*Aip*^*fl/fl*^;*Rag1*^*Cre/+*^) (referred to as *Aip*^*fl/fl*^ Cre^+^ mice). This resulted in deletion of *AIP* as determined by qPCR and western blot analysis (Figures S1A and S1B). These mice presented no spontaneous signs of pathology from birth to the age when they were used for experiments (9–12 weeks).

**Figure 1:**
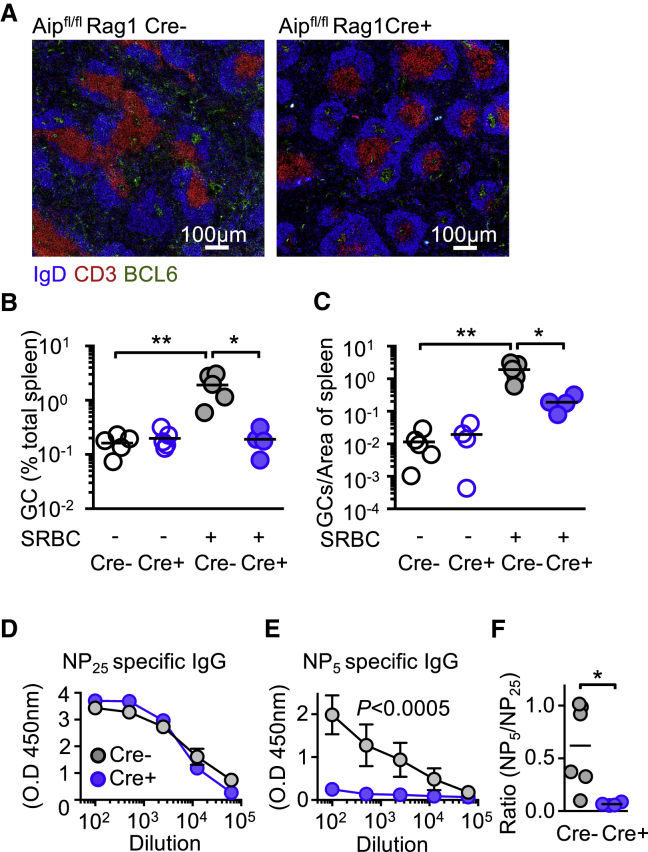
AIP Regulates Adaptive Immune Responses (A–C) *Aip*^*fl/fl*^ Cre^+^ (B) and Cre^−^ control (A) mice (Figures S1A and S1B) were immunized with sheep red blood cells (SRBCs), and 10 days later, the size (A and B) and number of germinal center (GC) B cells (BCL6^+^ area within the IgD^+^ follicle; A and C) was determined. *Aip*^*fl/fl*^ Cre^+^ mice and littermate controls were immunized with NP-KLH absorbed with aluminum hydroxide and examined 14 days after immunization. (D and E) Serum was examined for the ability to bind to antigen with a high-valence (low-affinity) (NP_25_) antigen (D) and a low-valence (high-affinity) (NP_5_) antigen (E). (F) The ratio of NP_5_:NP_25_ affinity antibodies from *Aip*^*fl/fl*^ Cre^+^ and littermate controls was determined. See also Figure S5. Scale bars, 100 μm. Results are from two or three independent experiments with two to four animals per experiment. ^∗^p < 0.05; ^∗∗^p < 0.01.

To gain insight into whether *Aip* deficiency affected adaptive immunity, *Aip*^*fl/fl*^ Cre^+^ and Cre^−^ littermate controls were immunized with sheep red blood cells (SRBCs) to induce a T cell-dependent immune response and sacrificed 10 days later (Sander et al., 2015). Analysis of the spleen revealed that in contrast to the *Aip*^*fl/fl*^ Cre^+^ animals, there was a significant increase of the GC area or number of GCs in Cre^−^ mouse spleen compared to *Aip*^*fl/fl*^ Cre^*+*^ spleens following SRBC immunization (p = 0.0146) (Figures 1A–1C).

We sought to determine whether *Aip*^*fl/fl*^ Cre^+^ mice had a defect in the ability to generate high-affinity antibodies. Mice were immunized with (4-hydroxy-3-nitrophenyl)-acetyl (NP)-keyhole limpet hemocyanin (KLH) precipitated to aluminum hydroxide (alum), and 2 weeks later, the capacity of serum immunoglobulins to bind to high-valency antigen (NP_25_) and low-valency antigen (NP_5_) was examined (Capasso et al., 2010). No difference was detected between the *Aip*^*fl/fl*^ Cre^+^ and Cre^−^ mice in the generation of low-affinity antibody against NP-KLH (Figure 1D). However, there was a significant reduction in the ability of *Aip*^*fl/fl*^ Cre^+^ mice to produce high-affinity antibody that could bind to NP_5_ (p = 0.0002) (Figure 1E), and consequently, the ratio between NP_5_ and NP_25_ specific antibodies between *Aip*^*fl/fl*^ Cre^+^ and Cre^−^ mice was low (p = 0.026) (Figure 1F).

### AIP Regulates GC Formation

**Figure 2:**
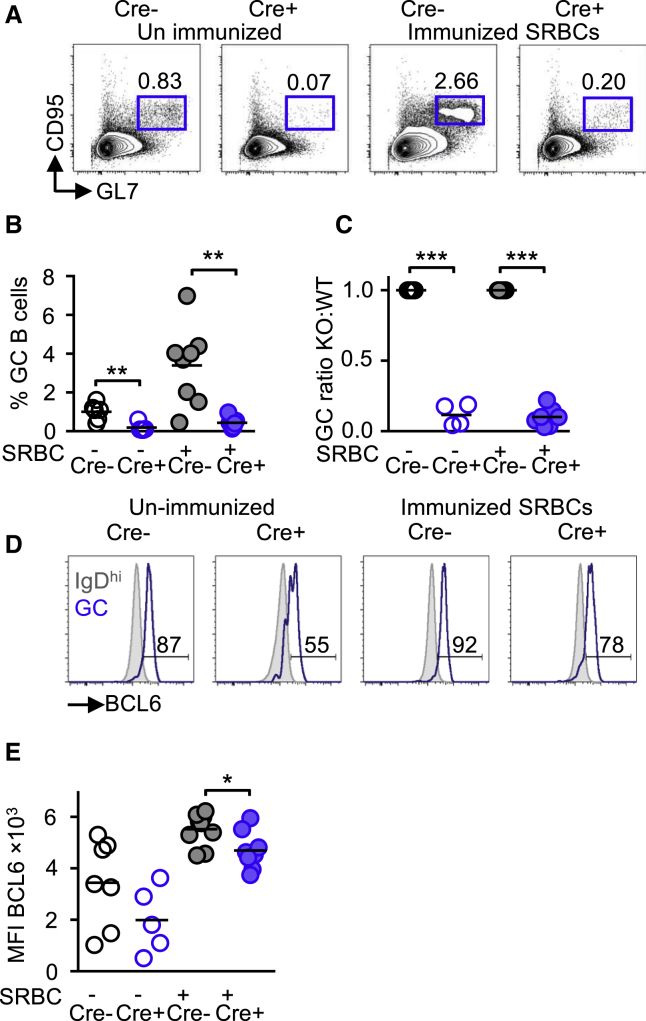
AIP Regulates GC Formation (A–C) GC B cells (B220^+^ GL7^+^ CD95^+^; in A) and percentage of GC B cells from *Aip*^*fl/fl*^ Cre^+^ mice (B; see also Figures S1C–S1F) and ratio of GCs between *Aip*^*fl/fl*^ Cre^+^ and Cre^−^ mice (C). (D and E) Lower expression of BCL6 as determined by flow cytometry (D) measuring the median fluorescent intensity (MFI; in E) (see also Figures S2–S4). Grey histograms represent biological control by gating on naive (IgD^hi^) B cells that do not express BCL6. Results are from two or three independent experiments with two to four animals per experiment. ^∗^p < 0.05; ^∗∗^p < 0.01; ^∗∗∗^p < 0.001.

The ability to make antibody responses against T cell-dependent antigens is dependent upon B cell differentiation into GC B cells (Victora and Nussenzweig, 2012). Nonimmunized *Aip*^*fl/fl*^ Cre^+^ had a significantly decreased percentage and ratio of GC B cells (GL7^+^ CD95^+^) (the gating strategy and phenotype are shown in Figures S1C–S1E) compared to littermate controls (p = 0.001) (Figures 2A–2D). Of particular interest was that *Aip*^*fl/fl*^ Cre^+^ GC B cells demonstrated a significantly lower expression and ratio of BCL6 compared to Cre^−^ GC B cells (p = 0.026) (Figures 2E and 2F).

Conversely, *Aip*^*fl/fl*^ Cre^+^ mice displayed a significant increase in the percentage of non-GC B cells in secondary lymphoid tissues (spleen and peripheral lymph nodes) along with increased circulating B cells (Figure S2A). Despite the significant increase in the number of B cells in the spleen, there was no difference in the cellularity of the spleen between Cre^−^ and *Aip*^*fl/fl*^ Cre^+^ mice, as *Aip*^*fl/fl*^ Cre^+^ mice also displayed a decrease in the total number of CD3^+^ T cells (Figures S2B–S2D). Similar to the spleen, we also observed decreased GC B cells and BCL6 expression in peripheral lymph nodes although there was an increase in GC B cells from Peyer’s patches in Cre^+^ mice (Figure S2A).

Differences in GC B cells may be the result of altered B cell development or the type of B cells produced. Examination of naive, marginal zone and follicular B cell subsets revealed no difference between Cre^−^ and *Aip*^*fl/fl*^ Cre^+^ mice (Figures S3A–S3D). BCL6 has been reported to contribute to B cell lymphopoiesis (Duy et al., 2010). Consequently, we examined developing B cell subsets in the bone marrow of Cre^−^ and *Aip*^*fl/fl*^ Cre^+^ mice but found no significant differences (Figure S3C). This indicated that the difference we were observing in *Aip*^*fl/fl*^ Cre^+^ B cells was restricted to GC B cells. However, examination of the bone marrow did reveal a lower percentage of IgM^+^ B220^hi^ B cells from *Aip*^*fl/fl*^ Cre^+^ mice (Figure S3D), suggesting that AIP impacted upon B cells within the bone marrow. We did not observe any changes in the immunoglobulin isotypes in serum or following *in vitro* stimulation of Cre^+^ B cells (Figure S3E), indicating that deficiency of AIP did not alter the production of antibodies or isotype switching.

As AIP was originally described as a co-chaperone for AHR (Meyer and Perdew, 1999), we examined *Ahr*^*fl/fl*^ mice crossed with *Rag1*^*Cre/+*^ mice to determine if they had a similar phenotype. In agreement with a publication studying the role of AHR in B cells (Villa et al., 2017), following SRBC immunization, we found no differences in GC B cells, BCL6 expression, or antibody production between *Ahr*^*fl/fl*^;*Rag1*^*Cre/+*^ mice and littermate control mice (Figures S4A–S4D). This suggested that AIP was acting independently of AHR to regulate GC B cells and BCL6.

Given the suggested role of AIP in regulating T cell-dependent antigen responses, we tested if there might be a reduced T response to T cell-independent antigens in *Aip*^*fl/fl*^ Cre^+^ mice. To test this, we immunized mice with an NP-Ficoll conjugate, a T cell-independent antigen that recruits marginal zone B cells to produce a low-affinity extra-follicular antibody response (García de Vinuesa et al., 1999). 10 days after immunization, we analyzed mice and measured the amount of NP-specific immunoglobulin M (IgM), IgG, and IgG3 in the serum by ELISA. *Aip*^*fl/fl*^ Cre^+^ mice could make NP-specific IgM (p = 0.0001) and IgG (p = 0.029) (Figures S5A and S5B), but the titer of the antibodies against NP was significantly lower compared to Cre^−^ mice. IgG3 is produced in response to T cell-independent antigens (García de Vinuesa et al., 1999), and *Aip*^*fl/fl*^ Cre^+^ mice made significantly less (p = 0.0031) IgG3 compared to Cre^−^ mice (Figure S5C), despite having normal levels of marginal zone B cells. Examination of the percentage of NP-specific B cells by flow cytometry showed that *Aip*^*fl/fl*^ Cre^+^ mice had significantly decreased percentage of NP-specific plasmablasts (B220^+/−^ CD138^+^) compared to Cre^−^ mice (p = 0.028) (Figures S5D and S5E). Together, these results demonstrated that AIP was required for the generation of development of antibody responses in both the GC and extra-follicular sites.

### AIP Regulates GC Organization and AKT Signaling in GC B Cells

**Figure 3:**
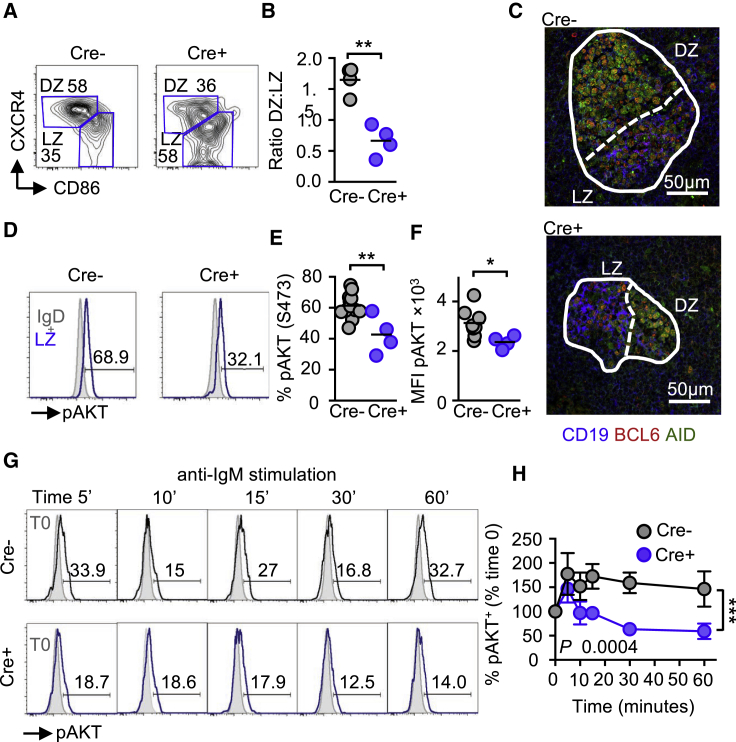
AIP Regulates GC Organization and AKT Signaling in GC B Cells (A) Expression of dark zone (DZ) (CXCR4^+^) and light zone (LZ) (CD86^+^) GC B cells from immunized *Aip*^*fl/fl*^ Cre^+^ and littermate controls. (B) Ratio of DZ and LZ GC B cells f. (C) Spleen sections from *Aip*^*fl/fl*^ Cre^+^ and Cre^−^ mice were analyzed by immunofluorescence for LZ CD19^+^ BCL6^+^ and DZ AID^+^ BCL6^+^ areas of GCs. (D–F) The phosphorylation of AKT (serine 473) was determined in LZ GC B cells from *Aip*^*fl/fl*^ Cre^+^ and littermate controls (D) and the percentage of pAKT (E) and MFI (F) determined. Grey histograms are gating on IgD^+^ naive B cells used as a biological control. (G and H) IgD^−^ B cells from Cre^+^ and Cre^−^ mice were stimulated with anti-IgM (10 μg/mL) and examined for the expression of phosphorylated AKT from 5 to 60 min post-stimulation (G) and the percentage increase from time zero determined (H). See also Figure S6. Grey histograms show expression at time zero (T0). Scale bars, 50 μm. Results are from two or three independent experiments with one or two mice per experimental group. ^∗^p < 0.05; ^∗∗^p < 0.01; ^∗∗∗^p < 0.001.

GC B cells undergo repeated cycles of rapid proliferation in the dark zone (DZ) followed by a resting state in the light zone (LZ) where the cells can reencounter, their antigen presented by follicular-dendritic cells. If the recognition is successful, then B cell progress to become memory B cells or plasmablasts or reenter the DZ to further increase their B cell receptor affinity (Allen et al., 2007, Mesin et al., 2016, Victora and Nussenzweig, 2012). DZ and LZ GC B cells can be distinguished by flow cytometry using the markers including CXCR4 (DZ) and CD86 (LZ) (Victora et al., 2010). Using this method, we analyzed GC B cells and found, in agreement with other studies, a ratio of ∼2 between DZ and LZ B cells in wild-type GC B cells. In contrast, there was a significantly reduced DZ/LZ ratio between *Aip*^*fl/fl*^ Cre^+^ and Cre^−^ mice (p = 0.001) (Figures 3A and 3B). Immunofluorescent analysis of Cre^−^ and *Aip*^*fl/fl*^ Cre^+^ spleen sections revealed that the area of the DZ (activation-induced deaminase [AID]^+^ and BCL6^+^ cells) was smaller in *Aip*^*fl/fl*^ Cre^+^ mice than in Cre^−^ mice (Figure 3C).

Phosphatidylinositol 3-kinase (PI3K) signaling contributes to the segregation of the DZ and LZ by phosphorylating AKT (serine 473) in LZ GC B cells, resulting in decreased expression of the chemokine receptor CXCR4 (Dominguez-Sola et al., 2015, Sander et al., 2015). We observed a significant decrease in phospho-AKT expression in LZ GC B cells from *Aip*^*fl/fl*^ Cre^+^ mice compared to Cre^−^ mice (p = 0.003) (Figures 3D–3F). AIP appeared to regulate the AKT pathway, as *in vitro* anti-IgM stimulation of *Aip*^*fl/fl*^ Cre^+^ B cells revealed that while AKT was rapidly phosphorylated in both *Aip*^*fl/fl*^ Cre^+^ and Cre^−^ deficient B cells following stimulation, AKT phosphorylation was subsequently reduced at a faster rate in Cre^+^ B cells than in Cre^−^ B cells (Figures 3G and 3H). AIP appeared to be specifically regulating AKT, as the extracellular signal regulated kinase (ERK) and SYK pathways were unaffected (Figures S6A and S6B).

### AIP Regulates GC B Cells Independently of T Cells

**Figure 4:**
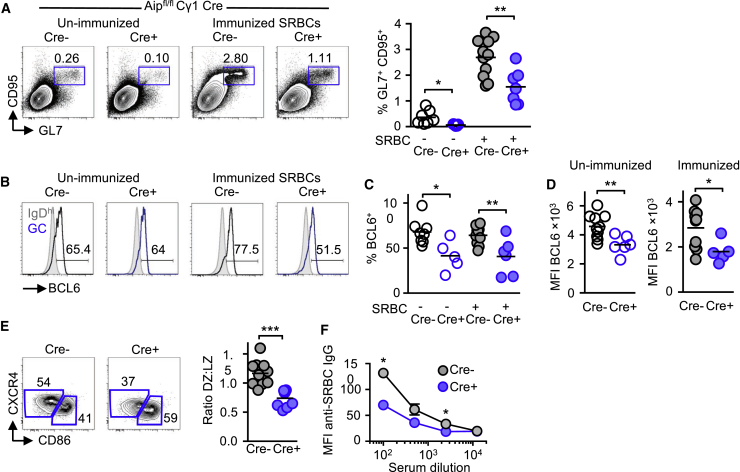
AIP Regulates GC B Cells Independently of T Cells (A and B) GC B cells (GL7^+^ CD95^+^) from *Aip*^*fl/fl*^ mice crossed with *Cγ1*^*Cre/+*^ mice and examined at baseline (open circles) or following immunization with 2 × 10^9^ SRBCs (filled circles) 10 days later (A) and the expression of BCL6 was assessed by flow cytometry (B). (C and D) The percentage of GC B cells (C) and MFI from baseline (unimmunized; D) and immunized *Aip*^*fl/fl*^; *Cγ1*^*Cre/+*^ mice was determined. (E) Expression and ratio of DZ (CXCR4^+^) and LZ (CD86^+^) GC B cells from immunized mice. (F) Serum anti-SRBC IgG determined by incubating serially diluting serum from Cre^−^ and Cre^+^ mice. Results are from two independent experiments with three mice per group. ^∗^p < 0.05; ^∗∗^p < 0.01.

As we had conditionally deleted *Aip* in both T and B cells, it raised the possibility that the defects observed in *Aip*-deficient B cells might be consequent to altered T cell help. We observed a decrease in T follicular helper (T_FH_) cells in *Aip*^*fl/fl*^ Cre^+^ mice (data not shown). T_FH_ cells are dependent on BCL6 for their development (Huang et al., 2013), and we observed a decrease in BCL6 expression in *Aip*^*fl/fl*^ Cre^+^ mice (data not shown). To address this issue, we crossed *Aip*^*fl/fl*^ mice with Cγ1-Cre mice to specifically delete *Aip* in GC B cells (Calado et al., 2012). Mice were immunized with SRBCs, and 10 days later, the percentage of GC (GL7^+^ CD95^+^) B cells and the expression of BCL6 were determined. As predicted, conditional deletion of *Aip* in GC B cells resulted in a lower percentage of GC B cells with a lower expression of BCL6 (Figures 4A–4D) and a decreased ratio of DZ to LZ GC B cells (Figure 4E) and decreased anti-SRBC IgG (Figure 4F). Together, these results indicated that AIP regulated GC B cells and BCL6 expression independently of T cells.

### AIP Protects BCL6 from FBXO11-Mediated Proteasomal Degradation

We sought to investigate the mechanism by which AIP sustains BCL6 expression in GC B cells. The E3 ligase containing F box protein O11 (FBXO11) has been shown to target BCL6 for ubiquitin-mediated proteasomal degradation (Duan et al., 2012). This observation attracted our interest as a potential mechanism by which AIP could regulate BCL6, as AIP had previously been shown to protect AHR from ubiquitin-mediated degradation (Kazlauskas et al., 2000) and AIP has recently been found to bind to FBXO3 (Hernández-Ramírez et al., 2016). We hypothesized that AIP was protecting BCL6 from ubiquitin-mediated proteasomal degradation via FBXO11.

**Figure 5:**
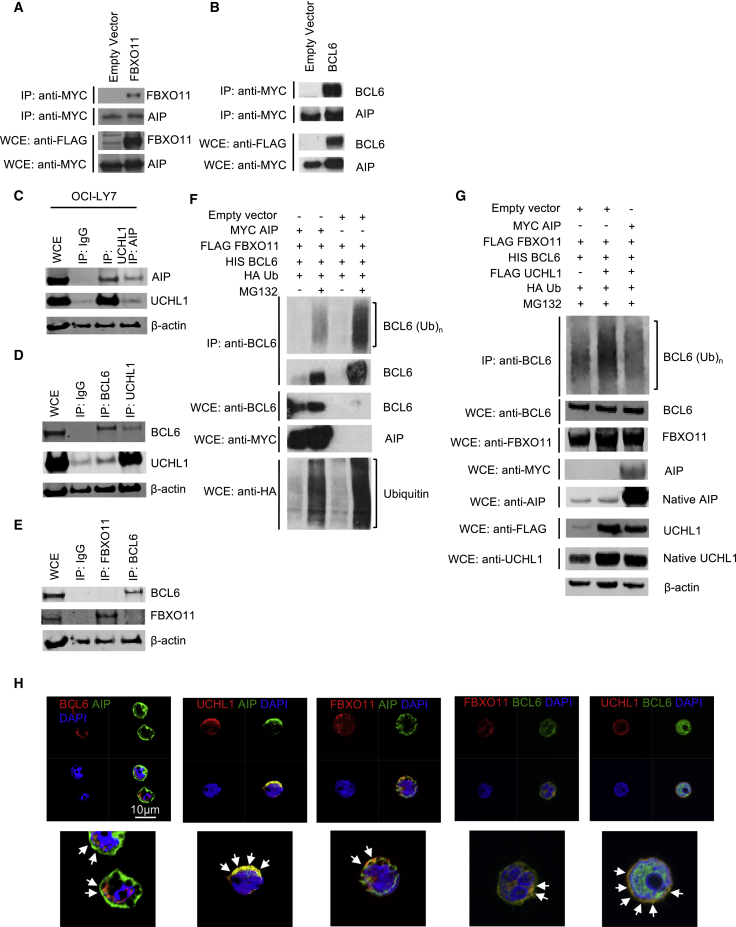
AIP Protects BCL6 from FBXO11-Mediated Proteasomal Degradation (A) HEK293T cells were transfected with either a FLAG-tagged FBXO11 or empty vector (EV-FLAG), together with MYC-tagged AIP. A majority of the whole-cell extracts (WCEs) were subjected to immunoprecipitation (IP) using FLAG antibody (rows 1 and 2), and the rest of the WCEs were used in immunoblotting (rows 3 and 4). (B) HEK293T cells were transfected with either FLAG-tagged BCL6 or EV-FLAG, together MYC-tagged AIP (Leontiou et al., 2008). A majority of the WCEs were subjected to IP using FLAG antibody (rows 1 and 2), the and the rest of the WCEs were used in immunoblotting (rows 3 and 4). (C) OCI-LY7 DLBCL cells were lysed and immunoprecipitated with antibodies to IgG, AIP, UCHL1 and immunoblottedfor AIP and UCHL1. (D) OCI-LY7 DLBCL cells were lysed and immunoprecipitated with antibodies to IgG, BCL6, UCHL1 and immunoblotted for BCL6 and UCHL1. (E) OCI-LY7 DLBCL cells were lysed and immunoprecipitated with antibodies to IgG, FBXO11 and BCL6 and immunoblotted for BCL6 and FBXO11. β-actin was used as a loading control. (F) HEK293T cells were transfected with MYC-tagged AIP or EV-MYC, FLAG-tagged FBXO11, HIS-tagged BCL6, and hemagglutinin (HA)-tagged ubiquitin. Where indicated, cells were treated with MG132 post-transfection for 2.5 h to inhibit proteasomal degradation. (G) HEK cells were transfected as in (F) and FLAG-tagged UCHL1. Cells were harvested and subjected to IP BCL6. Corresponding WCEs are shown. (H) OC1-LY7 cells were stained with AIP, BCL6, UCHL1, and FBXO11. DAPI was used as a nuclear stain. Arrowheads show areas of co-localization.

Immunoprecipitation (IP) analysis of HEK cells transfected with MYC-tagged AIP and FLAG-tagged FBXO11 revealed that AIP could bind to FBXO11 and BCL6, thereby revealing a potential mechanism of action (Figures 5A and 5B). Ubiquitin E3 ligases are often found in association with deubiquitinases (DUBs) of the same substrate (Komander et al., 2009). We therefore wanted to know if AIP associated with any DUBs that might regulate BCL6 expression. Mass-spectrometry analysis had revealed that AIP could bind to the DUB UCHL1 (Hernández-Ramírez et al., 2016). UCHL1 is induced in GC B cells and cooperates with BCL6 to promote a mouse model of lymphoma and is associated with an aggressive subset of diffuse large B cell lymphoma (DLBCL) (Bedekovics et al., 2016, Hussain et al., 2010). We hypothesized that AIP might support the expression of BCL6 by binding UCHL1 and support the deubiquitination of BCL6.

Using the functionally relevant DLBCL cell line OCI-LY7, we found that AIP could bind to UCHL1 (Figure 5C) and that UCHL1 could bind to BCL6 (Figure 5D), indicating that UCHL1 could be responsible for maintaining BCL6 expression. In agreement with Duan et al. (2012), we found that OCI-LY7 cells expressed FBXO11, although interestingly, using these cells, we found that FBXO11 could not directly bind to BCL6 (Figure 5E). To determine if FBXO11-mediated degradation of BCL6 could be modulated by AIP, we transfected HEK cells with epitope-tagged FBXO11, BCL6, AIP, and ubiquitin plasmids. The addition of AIP reduced FBXO11-mediated ubiquitin conjugation to BCL6. The absence of AIP resulted in an increase in BCL6 ubiquitination (in the presence of FBXO11) and subsequently decreased BCL6 expression, recapitulating what we observed in *Aip*-deficient B cells (Figure 5F).

BCL6 has not been described as being a substrate for UCHL1, so we tested whether UCHL1 could deubiquitinate BCL6. Transfection of HEK cells revealed that UCHL1 could deubiquitinate BCL6 only in the presence of AIP (Figure 5G), indicating that AIP regulated the function of UCHL1. To confirm the IP results, we performed confocal microscopy using the OC1-LY7 lymphoma cell line. AIP co-localized with BCL6, UCHL1, and FBXO11. BCL6 co-localized with FBXO11 and, to a greater extent, UCHL1 (Figure 5H). Together, these results revealed the mechanism by which AIP regulated BCL6 expression in GC B cells.

### AIP Is Overexpressed in Human DLBCLs

**Figure 6:**
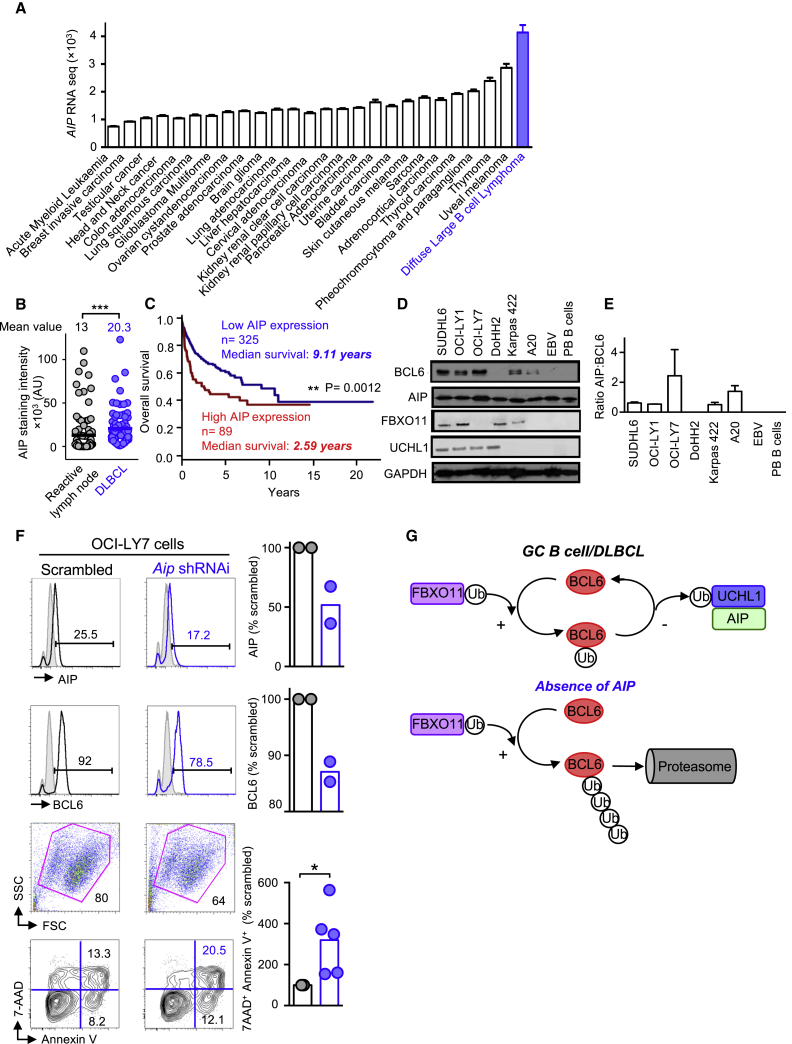
AIP Is Overexpressed in Human DLBCLs (A) *AIP* is expressed in many tumors, and *AIP* was found to be most highly expressed in DLBCLs. Data were obtained from http://www.ciobioportal.org. (B) AIP staining from reactive lymph nodes (n = 88 mean 13 ± 2.5) and DLBCL biopsies (n = 74 mean 20 ± 2.5) ^∗∗∗^p < 0.0001 (two-tailed Mann-Whitney U test). (C) Survival analysis of DLBCL patients with high and low AIP expression. Data obtained from http://www.genomicscape.com/. (D) BCL6 and AIP protein expression in DLBCL cell lines. (E) Ratio between AIP and BCL6 expression. EBV, Epstein-Barr-virus-infected B cells; PB, peripheral blood B cells. (F) Lentiviral delivery of scrambled or shRNAi against *Aip* to OC1-LY7 cells. Cells were examined 48 h after transfection, and the expression of AIP and BCL6 and cell viability were analyzed by flow cytometry. (G) Diagram showing the interaction between FBXO11, UCHL1, BCL6, and AIP. Under genotoxic environments found in GC B cells or DLBCL cells, AIP brings UCHL1 to deubiquitinate BCL6, thus maintaining its expression. In the absence of AIP, BCL6 gets ubiquitinated by FBXO11 and undergoes proteasomal degradation. ^∗∗∗^p < 0.0001 (a Mann-Whitney U test).

Examination of the cbioportal database for cancer genomics (http://www.cbioportal.org; Cerami et al., 2012) revealed that *AIP* expression was higher in DLBCLs than in other cancers and tumors (Figure 6A). Histological analysis of primary DLBCL biopsy samples revealed that AIP expression was significantly increased compared to control (reactive lymph node) samples (p < 0.0001) (Figure 6B). Data obtained from genomicscape (http://www.genomicscape.com/) revealed that DLBCL patients with high *AIP* expression had significantly (p = 0.002) reduced survival compared to those with low *AIP* expression, indicating that increased *AIP* expression contributed to the morbidity of DLBCL (Figure 6C). Western blot analysis revealed that AIP was expressed in a number of DLBCL cell lines and that its expression matched BCL6 expression (Figures 6D and 6E).

To determine if knockdown of *AIP* in lymphoma cells affects their viability, we performed lentiviral knockdown of AIP in OC1-LY7 DLBCL lymphoma cells. Knockdown of *AIP* in OC1-LY7 cells resulted in decreased AIP and BCL6 expression and decreased the viability of the DLBCL lymphoma cells (Figure 6F).

Based upon these results, we propose that AIP positively regulates BCL6 expression by binding to the DUB UCHL1, thereby preventing FBXO11-mediated ubiquitination of BCL6 and contributing to the maintenance of BCL6 expression in GC B cells and DLBCLs (Figure 6G).

## Discussion

Lymphocytes need to maintain cellular homeostasis as they navigate a range of genotoxic events and environments. A particularly challenging environment is within GCs, where B cells undergo rapid proliferation, class switching, and somatic hyper-mutation. How B cells preserve cellular homeostasis in this environment is still not completely clear.

We provide evidence that AIP is required for mounting immune responses against T cell-dependent and, to a lesser extent, T cell-independent antigens and the generation of high-affinity antibodies. Consequently, we found that *Aip*^*fl/fl*^ Cre^+^ mice and conditional deletion of *Aip* in GC B cells display a significant reduction in the percentage of GC B cells and decreased BCL6 expression, partly providing an explanation for impaired adaptive immune responses.

We focused on AIP regulation of BCL6, as it was an obvious starting candidate when we observed decreased GC B cells in *Aip*^*fl/fl*^ Cre^+^ mice. In line with our observations, *Bcl6*-deficient mice have impaired GC responses similar to the phenotype we observed (Huang et al., 2013, Ye et al., 1997). However, not all the effects that we see in our *Aip*^*fl/fl*^ Cre^+^ mice can be attributed to decreased expression of BCL6; for example, reduced extra-follicular immune responses and reduced AKT phosphorylation are not features of *Bcl6*-deficient mice, and to our knowledge, BCL6 is not known to regulate the migration of B cells.

*Aip*-deficient B cells initially phosphorylated AKT similar to wild-type B cells; however, AKT phosphorylation was rapidly lost in *Aip*-deficient B cells in contrast to wild-type B cells. We have observed similar defects in AKT signaling following insulin stimulation in GH3 pituitary cells with lentiviral *Aip* knockdown (M.K. and O.H., unpublished data). HSP90 has been found to regulate AKT expression and phosphorylation (Sato et al., 2000), and UCHL1 has also been found to regulate AKT signaling (Hussain et al., 2010), and we are currently determining how AIP regulates AKT signaling.

AIP is a co-chaperone for AHR (Kazlauskas et al., 2000, Lees et al., 2003). We were therefore surprised to find that conditional deletion of *Ahr* in B and T cells did not match the phenotype we observed in *Aip*^*fl/fl*^ Cre^+^ mice. This indicated that AIP was acting independently of AHR to regulate GC B cells. This finding is supported by Villa et al., who showed that AHR supported B cell proliferation but was not necessary for T cell-dependent or independent immune responses (Villa et al., 2017). However, a recent publication reports that AHR was important in suppressing T cell-dependent and T cell-independent immune responses (Vaidyanathan et al., 2017). The reasons for the different responses from *Ahr* null B cells are not clear at present.

As BCL6 is an important oncoprotein, there is considerable interest in how it is regulated. BCL6 expression can be controlled via post-translational mechanisms, including phosphorylation (Niu et al., 1998), mRNA stability, and nuclear export by eIF4Ae (eukaryotic translation initiation factor 4E) (Culjkovic-Kraljacic et al., 2016), in response to DNA damage (Phan et al., 2007) and stabilization by HSP90 (Cerchietti et al., 2009). Mitogen-activated protein kinase (MAPK) signaling following B cell receptor stimulation has been shown to be involved in the degradation of BCL6 (Niu et al., 1998). We saw no evidence of increased ERK signaling in *Aip* null B cells compared to wild-type B cells following anti-IgM stimulation, suggesting that this is not the mechanism by which AIP regulated BCL6 expression.

FBXO11 has been found to target BCL6 for ubiquitin-mediated degradation and mutations in FBXO11 associated with lymphoma (Duan et al., 2012). We found that AIP and the FBXO11-containing E3 ligase complex co-immunoprecipitation (co-IP), and we demonstrated that AIP inhibited FBXO11-mediated ubiquitination of BCL6. Intriguingly, mice deficient for FBXO11 in their B cells show a phenotype that is the opposite of ours; namely, an increased percentage of GC B cells and DZ GC B cells. These mice are also more prone to developing lymphoproliferative disease (Schneider et al., 2016). The DUB UCHL1 has been found to be expressed in GC B cells and lymphoma cells (Hussain et al., 2010), but the functional relevance of this expression of UCHL1 has not been determined. We found that UCHL1 could deubiquitinate BCL6, but only in the presence of AIP, indicating that AIP regulated the function of UCHL1. The precise mechanism by which AIP regulates UCHL1 will be the focus of future research, but DUBs often require co-factors to function (Komander et al., 2009).

AIP is a co-chaperone of HSP90 that can bind to proteins that control GC B cell phenotype, including c-MYC, STAT5, and nuclear factor κB (NF-κB), in addition to BCL6 (Cerchietti et al., 2009, Hertlein et al., 2010, Paul et al., 2013, Weigert et al., 2012). We have not ruled out the possibility that AIP can regulate these molecules, as these might contribute to the phenotype we observe in *Aip*-deficient B cells. This is the focus of ongoing research.

Ubiquitin E3 ligases often work in close proximity to DUBs to provide precise control over substrate proteins (Komander et al., 2009). Increased expression of the DUB UCHL1 has been associated with development of lymphoma and is a positive regulator of AKT signaling (Bedekovics et al., 2016, Hussain et al., 2010), but in B cells, its substrates and how it is regulated have not been identified. We found UCHL1 to be a binding partner of AIP and BCL6, indicating that it might function to help maintain BCL6 expression in GC and DLBCL cells.

There is significant evidence that chaperones are important regulators of protein quality control, helping ubiquitin E3 ligases and DUBs recognize their target proteins (Kriegenburg et al., 2012, Lee et al., 2014, Manjarrez et al., 2014, Morales and Perdew, 2007, Perrody et al., 2016). Chaperone molecules contribute to the pathobiology of cancers by protecting labile proteins from being degraded and supporting signaling pathways that cancer cells are dependent upon (Polier et al., 2013, Whitesell and Lindquist, 2005, Zong et al., 2015). We found AIP to be predominantly expressed in DLBCL compared to other tumors and overexpressed in DLBCL biopsy samples compared to tonsil tissue, indicating that AIP might contribute to DLBCL pathology. Knockdown of *AIP* in DLBCL cells resulted in decreased viability of DLBCL cells, indicating that targeting AIP could be used as a potential treatment for DLBCL.

How BCL6 expression is maintained is still not completely understood. The data presented here reveal that AIP is a positive regulator of BCL6 protein expression, which is commonly upregulated in B cell lymphomas, and show that AIP binds to an E3 ligase (FBXO11) and a DUB (UCHL1), both of which have been associated with DLBCL pathobiology. Therefore, AIP is a potential therapeutic target to treat DLBCLs.

## STAR★Methods

### Key Resources Table

**Table:** 

REAGENT or RESOURCE	SOURCE	IDENTIFIER
**Antibodies**		

CD16/32 Supernatant harvested from hybridoma	Raulet Lab, UC, Berkeley	N/A
B220 FITC clone RA3-6B2	Biolegend	AB_312990
CD4 APC clone GK1.5	Biolegend	AB_312696
CD3e AF700 clone145-2C11	Thermo Fisher Scientific (eBioscience)	AB_837094
CD21/35 clone eBio8D9	Thermo Fisher Scientific (eBioscience)	AB_10855041
CD23 PE-Cy7 clone B3B4	Biolegend	AB_2103037
CD38 BV421 clone 90	Thermo Fisher Scientific (eBioscience)	AB_11218302
CD95 PE clone 15A7	Thermo Fisher Scientific (eBioscience)	AB_465788
CD83 eFluor450 clone Michel-19	Biolegend	AB_2566123
CD138 PE clone 281-2	Biolegend	AB_10915989
IgD APC clone11-26c	Thermo Fisher Scientific (eBioscience)	AB_10598660
IgM eFluor 450 clone eB121-15F9	Thermo Fisher Scientific (eBioscience)	AB_10671539
CD24 APC clone M1/69	Thermo Fisher Scientific (eBioscience)	AB_2534261
CD43 PE clone eBioR2/60	Thermo Fisher Scientific (eBioscience)	AB_465659
CXCR4 PerCP clone L276F12	Biolegend	AB_2562786
Rat anti CD19 eFlour660 clone eBio1D3	Thermo Fisher Scientific (eBioscience)	AB_657650
CD86 PE-Cy7 clone GL1	Thermo Fisher Scientific (eBioscience)	AB_2573372
GL-7 Pacific Blue clone GL-7	Biolegend	AB_2563291
AIP	Novus Biologicals	AB_10002466
BCL6 PerCP clone BCL-DWN	Thermo Fisher Scientific (eBioscience)	AB_2573767
BCL6	CST	AB_10949970
FBXO11	Bethyl Laboratories	AB_890603
UCHL1	Proteintech	AB_2210497
pAKT eFluor 450 clone SDRNR	Thermo (eBioscience)	AB_2574125
p-ERK PE	Thermo Fisher Scientific (eBioscience)	AB_2572695
p-SYK PE	Thermo Fisher Scientific (eBioscience)	AB_2572675
Anti-mouse IgG biotin	Thermo Fisher Scientific (eBioscience)	AB_466650
Anti-mouse IgM	Jackson Laboratory	AB_2340761
Anti-mouse CD40 clone 1C10	Biolegend	AB_312942
Rabbit anti BCL6 clone N-3	Santa Cruz	AB_2063450
Rat anti-mouse IgD APC clone 11-26c.2a	BD Biosciences	AB_10612002
Anti-mouse IgM FITC	Southern Biotech	AB_2687524
Goat goat anti-mouse IgG_1_ Alexa Flour 647	Life Technologies	AB_141658
Goat anti-mouse IgG_2a_ Alexa Flour 647	Life Technologies	AB_141698

**Bacterial and Virus Strains**		

pHIV1 lentivirus plasmid	Barts Cancer Institute	N/A
pVSV-G lentivirus plasmid	Barts Cancer Institute	N/A
Biological Samples		
Human DLBCL tissue microarray for 33 patients	Barts Cancer Institute Tissue Bank	N/A

**Chemicals, Peptides, and Recombinant Proteins**		

Sheep Red Blood Cells	TSC Biosciences	Cat # SB069
NP-PE	Biosearch Technologies	Cat # N-5070-1
NP-BSA	Biosearch Technologies	Cat # N-5050XL-10
NP-Keyhole Limpet Hemocyanin	Biosearch Technologies	Cat # N-5060-5
Recombinant mouse IL-4	Peprotech	Cat # 214-14

**Experimental Models: Cell Lines**		

HEK293T	ATCC	CRL-3216
OC1-LY7	ATCC	ACC688
SUDHL6	Barts Cancer Institute	N/A
OC1-LY1	Barts Cancer Institute	N/A
DoHH2	Barts Cancer Institute	N/A
Karpas422	Barts Cancer Institute	N/A
A20	Barts Cancer Institute	N/A
EBV of healthy control	Korbonits Lab (Hernández-Ramírez et al., 2016)	N/A

**Experimental Models: Organisms/Strains**		

Aip flox/flox mice	Jackson Laboratories	013195
Ahr flox/flox mice	Stockinger Lab, Crick Institute, London (Li et al., 2011)	N/A
Rag1 Cre mice	Stockinger Lab, Crick Institute, London (Li et al., 2011)	N/A
Cγ1 Cre mice	Calado Lab, Crick Institute, London (Calado et al., 2012)	N/A

**Oligonucleotides**		

*Rag1* KI CRE F: 5′-TTTGTTTTTGTTTGCTTGTTTGA	Sigma	N/A
*Rag1* WT R: 5′-ATCCTTCTCCTTCTGTGCTTCTT	Sigma	N/A
*Rag1* KI CRE (V2): 5′-AATGTTGCTGGATAGTTT TTACTGC	Sigma	N/A
*Cγ1* IgG1 Kpn1 (WT): 5′ -TGTTGGGACAAACG AGCAATC	Sigma	N/A
*Cγ*1 Cre Cre13 (CRE) 5′-GGTGGCTGGACCAA TGTAAATA	Sigma	N/A
*Cγ1* Cre IgG1Rev (Common) 5′-GTCATGGCAAT GCCAAGGTCGCTAG	Sigma	N/A
*Aip*: F 5′-CAATCCCCCACTGTCACTT	Sigma	N/A
*Aip*: R- 5′-TCACCCCTCCCACTGACTAC	Sigma	N/A
*Aip Smart vector lentiviral shRNA*	Dharmacon	N/A

**Recombinant DNA**		

AIP-MYC pcDNA3.1	Korbonits Lab (Leontiou et al., 2008)	N/A
BCL6-FLAG pcDNA3.1	SinoBiological	Cat # NM_138931
FBXO11-FLAG pCMV3	SinoBiological	Cat # BC130445
Ub-HA pRK5	Nightingale Lab (QMUL, London)	N/A
UCHL1-FLAG	SinoBiological	Cat # NM_004181.4

**Software and Algorithms**		

PRISM version 6	Graphpad.com	N/A
FlowJo Version 9.3.1	Tree Star	N/A
Ariol Software	Leica Biosystems	N/A

### Contact for Reagent and Resource Sharing

Further information and requests for resources and reagents should be directed to and will be fulfilled by the Lead Contact, Oliver Haworth (o.haworth@westminster.ac.uk).

### Experimental Model and Subject Details

#### Mice

*Aip*^*fl/fl*^ (Jackson laboratories) and *Ahr*^*fl/fl*^ mice (Li et al., 2011) were crossed with *Rag1*^*Cre/+*^ mice (a kind gift from Brigitta Stockinger, Crick Institute) to specifically delete *Aip* in *Rag1* expressing cells (T and B cells) as previously described (Li et al., 2011), and Cγ1^Cre/+^ mice (a kind gift from Dinis Calado, Crick Institute; Calado et al., 2012) to specifically delete *Aip* in GC B cells. Male and female mice were born to expected Mendelian ratios and had no signs of any abnormalities until they were used at 9-11 weeks of age. Male and female mice were randomly assigned to experimental groups. All mice were maintained in a barrier facility and all experiments were approved and performed adhered to Home Office regulations (Guidance on the Operation of Animals, Scientific Procedures Act, 1986) and Queen Mary University of London ethics committee on the use of animals for research. Genotyping was performed using the primers listed in Key Resources Table.

#### Cell lines

HEK293T (ATCC), OC1-LY7 (ATCC), SUDHL6, OC1-LY1, DoHH2, Karpas422, A20 (Barts Cancer Institute), human EBV (Hernández-Ramírez et al., 2016)

#### Tissue array

Human DLBCL tissue microarray (33 patients, age range 23-86y). Sample collection approved by the local Human Ethics Committee.

### Method Details

#### Immunizations

Mice were immunized in the peritoneum with either 2 × 10^9^ fresh SRBCs (TSC Biosciences Ltd, UK) and 10 days later sacrificed as in Calado et al. (2012). NP (4-hydroxy-3-nitrophenyl-acetyl) conjugated to Keyhole Limpet Hemocyanin (KLH) or to Ficoll (Biosearch Technologies, Petaluma, USA) and examined 14 days later (Capasso et al., 2010, García de Vinuesa et al., 1999).

#### Collection and staining of tissues

Mouse tissues were stained using primary antibodies (Key Resources Table). Nuclei were detected using Hoechst 33258 and FITC was amplified using goat anti-FITC Alexa Flour 488 (Life Technologies). For AID staining a tertiary step of donkey anti-goat FITC (Jackson ImmunoResearch) was used. Slides were imaged using the Zeiss LSM 780 Confocal Microscope and Zeiss Axio Scan.Z1. Image analysis was performed using Zeiss Zen 2012 software to determine germinal center, follicular and spleen area. Plasma cells were counted by hand.

#### IgM stimulation of B cells

Spleen cells were stimulated with anti-mouse IgM (10μg/ml) (Jackson ImmunoResearch). At the end of the experiment, cells were immediately put on ice, fixed and permeabilized using and analyzed by flow cytometry for phosphorylated signaling molecules.

#### *In vitro* cell culture of B cells

B cells were isolated by MACS isolation or cell sorting and 10^5^/well were stimulated with 1 μg/ml anti-CD40 (Jackson ImmunoResearch) and 25ng/ml IL-4 (eBioscience) and the cells examined 38, 72 and 96 hours after stimulation.

#### Flow cytometry

Single-cell suspensions of tissues were prepared by passing spleen and lymph nodes through a 70 μm cell strainer and incubated with anti-CD16/32 (2.4G2) to block non-specific binding and then stained with antibodies (Key Resources Table). Cells were fixed and permeabilized using the Biolegend intracellular staining and permeabilization buffer according to the manufacturer’s instructions. Samples were acquired on an LSR Fortessa (BD) and the data analyzed using FlowJo software version 9.3.1 (Tree Star, Inc, Ashland, USA).

#### Detection of sheep red blood cell-specific antibodies

This was performed as described in Calado et al. (2012). Serum from mice was serially diluted and incubated with SRBC for 20 minutes on ice washing in cold phosphate-buffered saline and staining the SRBCs with a phycoerythrin (FITC)-conjugated anti-mouse IgG antibody and the samples were analyzed by flow cytometry.

#### Western blotting

Cells were re-suspended in cold RIPA lysis buffer (50mM Tris-HCl, pH 8.0, 10% sodium deoxycholate, 10% SDS, 150mM NaCl, 5mM EDTA, pH 8.0), supplemented with a mixture of protease inhibitors (1mM phenylmethylsulphonyl fluoride, 10mM sodium fluoride, 1mM sodium vanadate, 1mg/mL Leupeptin, 2mg/ml Aprotinin, 1M B-glycer). Samples were separated by standard SDS-polyacrylamide gel electrophoresis (SDS–PAGE). Protein extracts (40 μg total protein/lane) were fractionated on 8% SDS polyacrylamide gels and transferred to nitrocellulose membranes before being incubated with primary antibodies.

#### Immuno-precipitation

HEK293T cells (ATCC) (0.5 × 10^6^) were plated in 10cm dishes and the next day transfected with AIP and BCL6 epitope tagged plasmids using Fugene (Promega) according to the manufacturer’s instructions. The next day, cells were washed with 1ml phosphate-buffered saline and lysed using lysis buffer (5M NaCl, 1M Tris, 75% glycerol, 0.5M, EDTA, NP-40, sodium fluoride, dH_2_O and sodium vanadate). Subsequently, the cell lysates underwent immune-precipitation executed under cold conditions (4°C) throughout. Cell lysates were subjected to an overnight incubation on a rotator with the primary antibody followed by a two-hour incubation with 50 μL of protein A and G agarose beads. Pellets were washed with 500 μL lysis buffer five times, re-suspended in 40 μL lysis buffer and 10 μL 4 × SDS sample buffer. Samples were boiled at 95°C for 5 minutes and left to cool on ice to proceed with SDS-PAGE for immuno-blotting.

#### AIP staining of DLBCL tissue biopsies

Tissue microarrays containing either reactive lymph node (tonsil) or DLBCL samples from the Bart’s Cancer Institute tissue bank were immuno-stained for AIP and tissue microarrays images were taken using an Olympus BX61 microscope and analyzed using Ariol software for the staining intensity of AIP.

#### Lentivirus production and transfection of B cells

Lentivirus was produced using the protocol described in Kutner et al. (2009) in accordance with institutional guidelines using lentivirus. ShRNAi sequences against human *AIP* were purchased from Dharmacon/GE Life sciences. Early passage OC1-LY7 cells (less than passage 10) (ATCC) grown in Iscove’s Modified Dulbecco’s Medium supplemented with 20% FBS, were transduced with lentivirus containing a scrambled sequence or shRNAi against *AIP* using the method described by Boulianne et al. (2017). 2.5 × 10^5^ OC1-LY7 cells were spin-oculated with 50 μL of lentivirus supernatants supplemented with 5mg/ml polybrene (Sigma) and spun for 90 minutes at 2300 rpm. After 4 hours, media was replaced with fresh media (Iscove’s modified Dulbecco’s Medium supplemented with 10% FBS) and 24 hours later the cells were examined. Transfection efficiency using green fluorescent protein was shown to be ∼85%.

### Quantification and Statistical Analysis

Statistical analysis was performed using Prism software (Version 6). Un-paired two-tailed Student’s t test, Mann-Whitney test and 2-way ANOVA test were used as appropriate. Significance was taken as p < 0.05. Statistical details of the experiments can be found in the Figure Legends and Results. Data are plotted as mean ± SEM with *n* the number of biological replicates. On figures significance is marked as ^∗^ p < 0.05, ^∗∗^ p < 0.01, ^∗∗∗^p < 0.001.

## Supplemental Information

Document S1. Figures S1–S6

Document S2. Article plus Supplemental Information
