# Supplementary material for: Aryl Hydrocarbon Receptor Interacting Protein Maintains Germinal Center B Cells through Suppression of BCL6 Degradation
Source: Cell Rep. 2019 Apr 30;27(5):1461–1471.e4. doi: 10.1016/j.celrep.2019.04.014 (PMC6506688; doi:10.1016/j.celrep.2019.04.014)
Supplement: Document S1. Figures S1–S6 [file mmc1.pdf]

Cell Reports, Volume *27*

Supplemental Information

Aryl Hydrocarbon Receptor Interacting Protein Maintains Germinal Center B Cells through Suppression of BCL6 Degradation

Dijue Sun, Urszula Stopka-Farooqui, Sayka Barry, Ezra Aksoy, Gregory Parsonage, Anna Vossenkämper, Melania Capasso, Xinyu Wan, Sherine Norris, Jennifer L. Marshall, Andrew Clear, John Gribben, Thomas T. MacDonald, Christopher D. Buckley, Márta Korbonits, and Oliver Haworth

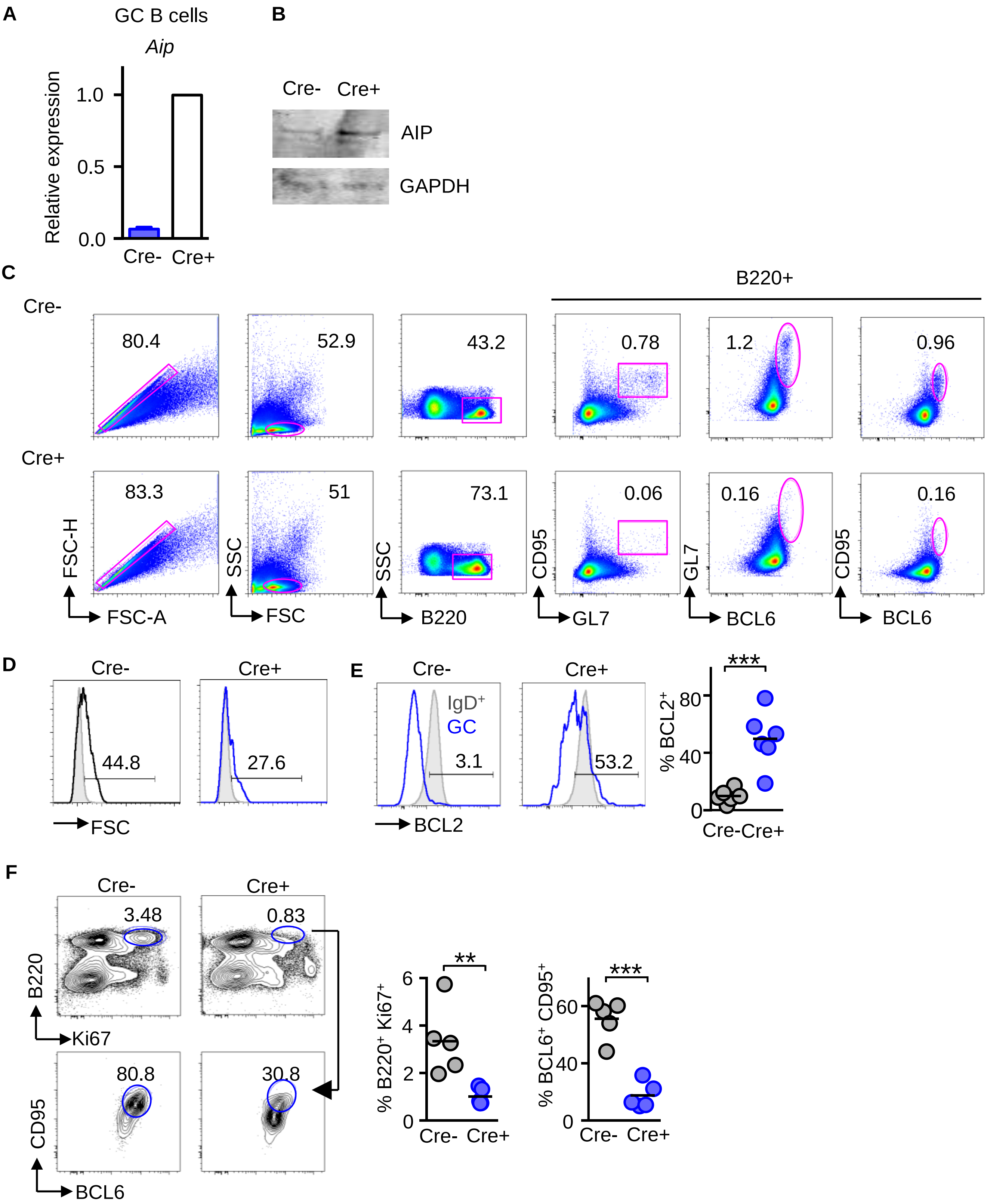

Figure S1. RAG1 Cre deletion of Aip and phenotype of GC B cells; related to Figure 1
(**A**) qPCR and (**B**) Western blot analysis of Aip from GC B cells from *Aip*$^{fl/fl}$ Cre^{+} and *Aip*$^{fl/fl}$ Cre^{-} mice. Data are plotted as mean ± SEM. (**C**) gating strategy to identify and analyze GC B cells. (**D**) size (FSC), (**E**) BCL2 expression, (**F**) proliferation of GC B cells from Cre^{-} and Cre^{+} mice.

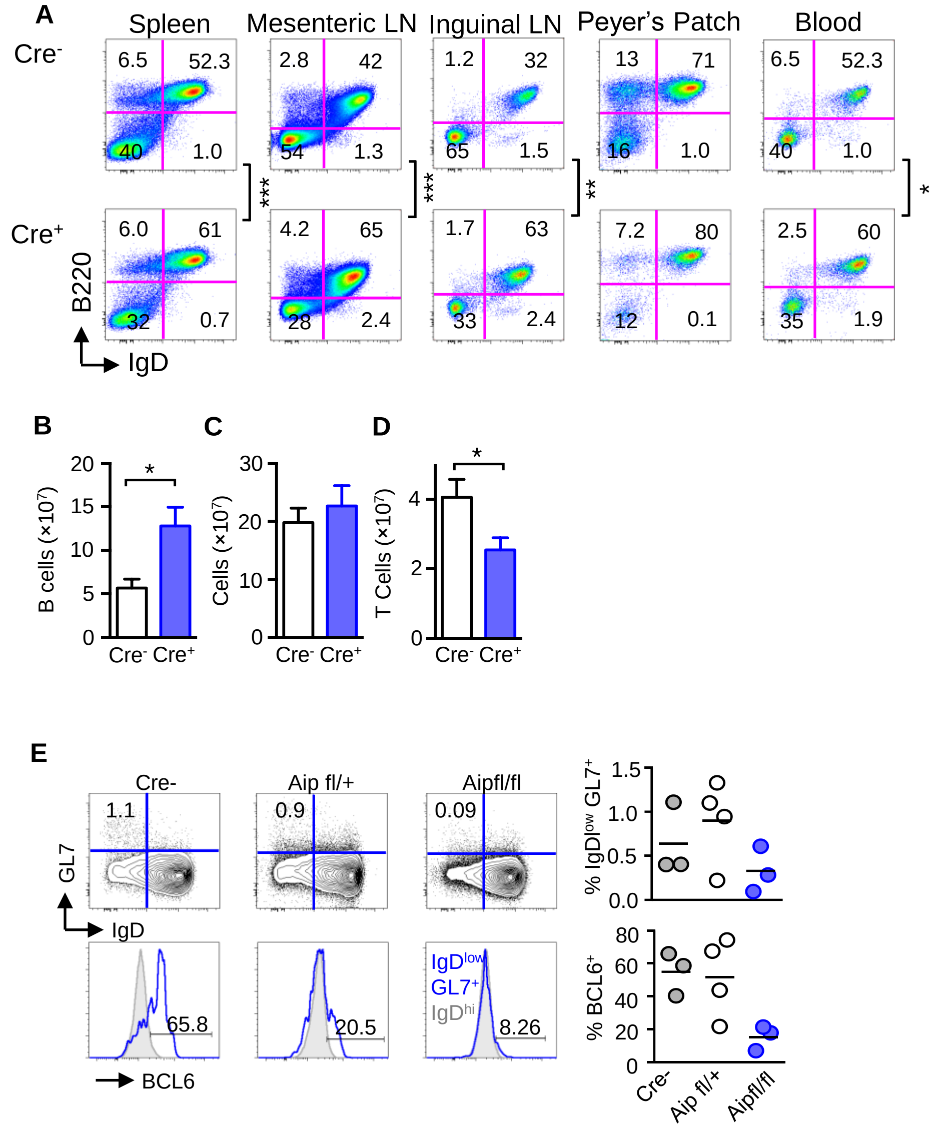

Figure S2. Increased B cells in the periphery of *Aip*$^{fl/fl}$ Cre^{+} mice; related to Figure 2

(**A**) Percentage of B cells in spleen, lymph nodes and circulating blood of B220^{+} IgD^{+} B cells from *Aip*$^{fl/fl}$ Cre^{+} mice compared to WT mice. Data shown as percentage ± SEM. Total number of B220^{+} IgD^{+} B cells (**B**) and total number of splenocytes (**C**). Total number of CD3^{+} T cells (**D**). Results are from 4-5 independent experiments, 2-3 mice per experimental group.

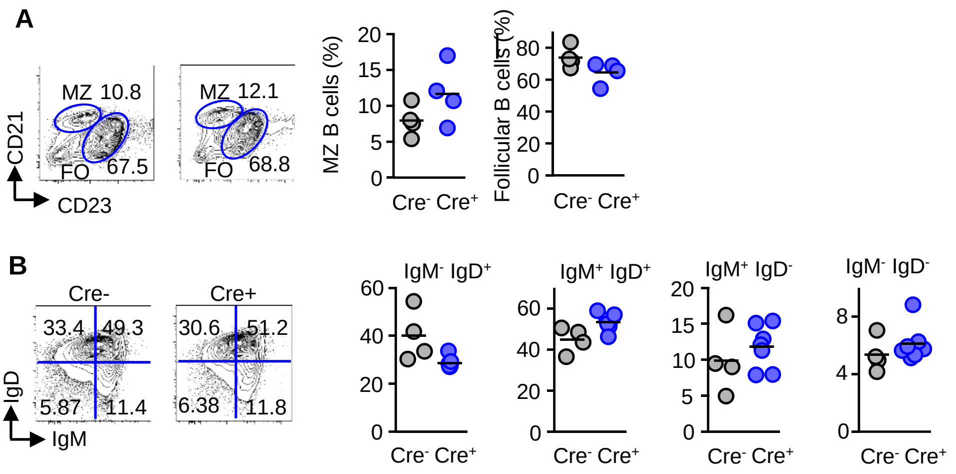

C

	ProB PreB	Pre B Pro B	Transitional	Early mature	Late mature
**Phenotype**	CD43+ CD24+ ⇨	IgM- IgD- ⇨	IgM+ IgD- ⇨	IgM+ IgD+ ⇨	IgM- IgD+
**RAG-1 expression**	+	+/-	low	-	-

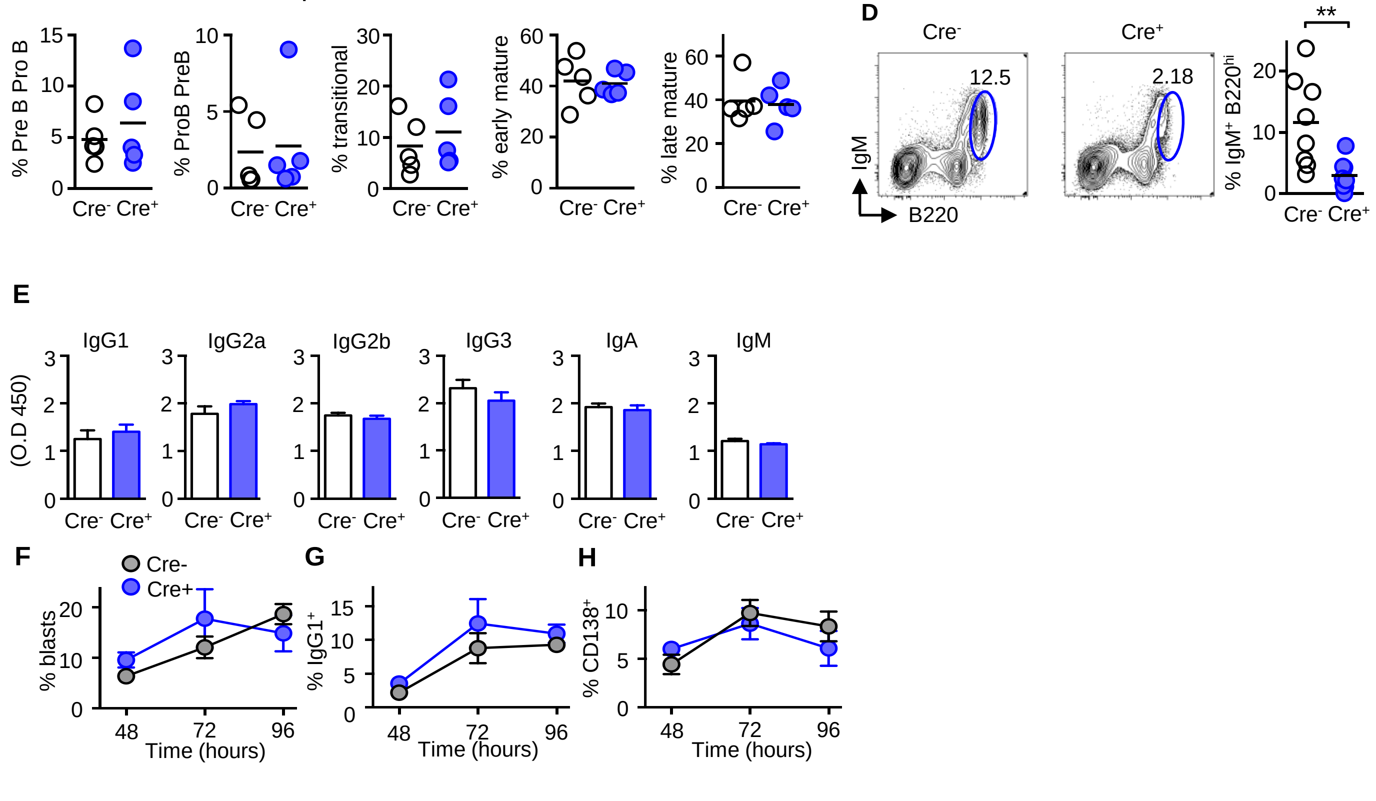

(legend on next page)

Figure S3. No difference in Marginal Zone or Follicular B cells or B cell development in *Aip*$^{fl/fl}$ Cre$^+$ mice; related to Figure 2

(**A**) Marginal zone (MZ) (CD21$^+$ CD23$^-$) and Follicular (FO) CD21$^-$ CD23$^+$) B cells were analysed by flow cytometry. (**B**) Expression of IgM or IgD on B cells from WT and *Aip*$^{fl/fl}$ Cre$^+$ mice. (**C**) Bone marrow from the femurs of Aip$^{fl/fl}$ Cre$^+$ and Cre$^-$ mice and were analyzed for different stages of B cell development by flow cytometry. B220$^+$ cells were stained with CD43 and CD24. The CD24$^+$ cells were divided into different sub-populations depending upon their expression of IgM and IgD. (**D**) Bone marrow from the femurs of *Aip*$^{fl/fl}$ Cre$^+$ and Cre$^-$ mice and were analyzed for B220^{hi} IgM$^{+/-}$ B cells by flow cytometry. There was a significant reduction in the percentage of B220^{hi} IgM$^+$ B cells from *Aip*$^{fl/fl}$ Cre$^+$ mice compared to Cre$^-$ mice. (**E**) serum from Cre- and Cre+ mice were examined by ELISA for immunoglobulin isotypes. B cells from Cre- and Cre+ were isolated and stimulated in vitro in the presence of anti-IgM, anti-CD40 and IL-4 and the percentage of proliferating cells (blasts) (**F**), IgG1 expression (**G**) and CD138 (**H**) examined at 48, 72 and 96 hours post stimulation by flow cytometry. 2-3 independent experiments, Results are from 2-3 mice per experimental group.

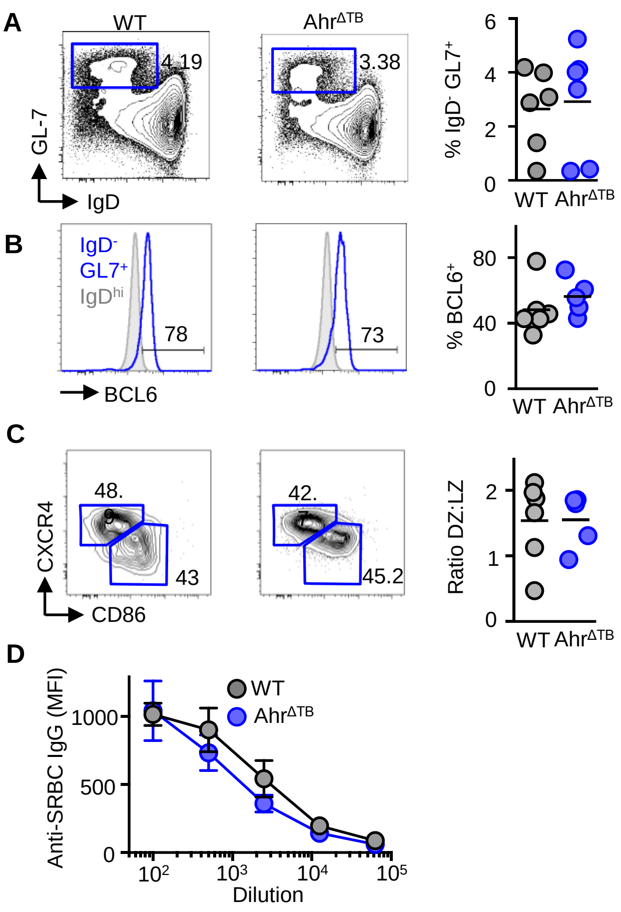

Figure S4. *AHR$^{\Delta TB}$* mice have normal GC B cells and immune responses towards SRBCs; related to Figure 2

(**A**) *AHR$^{\Delta TB}$* and Cre^{-} mice were immunized with SRBCs and the percentage of GC B cells (B220^{+} IgD^{-} GL-7^{+}) examined. (**B**) BCL6 expression determined from *AHR$^{-/-}$* and *AHR$^{+/+}$* IgD^{-} B cells. (**C**) DZ (CXCR4^{+}) and LZ (CD86^{+}) GC B cells from *AHR$^{\Delta TB}$* and Cre^{-} B cells and the DZ/LZ ratio. (**D**) Serum from *AHR$^{\Delta TB}$* and Cre^{-} mice immunized with SRBCs were serially diluted and incubated with SRBCs and the amount of IgG bound determined by flow cytometry. Results are from two independent experiments, 2-3 mice per experimental group. Data are plotted as mean ± SEM.

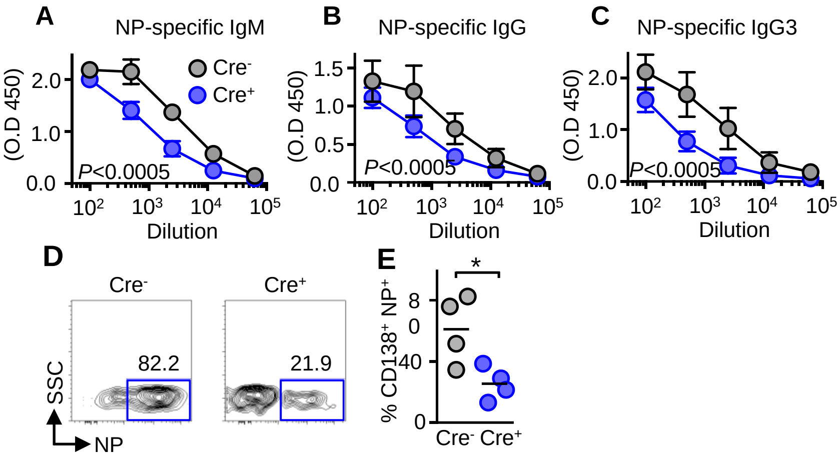

Figure S5. *Aip*$^{fl/fl}$ Cre^{+} mice decreased extra-follicular immune responses; related to Figure 1
Aip$^{fl/fl}$ Cre^{+} and Cre^{-} mice were immunized with NP-Ficoll and analyzed 14 days later NP-specific serum (**A**) IgM, (**B**) IgG and (**C**) IgG3 analyzed by ELISA. Data are plotted as mean ± SEM. (**D-E**) Percentage of NP-specific plasmablasts (B220$^{+/-}$ CD138^{+}) analyzed by flow cytometry. Representative FACS plots from at least 2 independent experiments, 2-3 mice per experimental group.

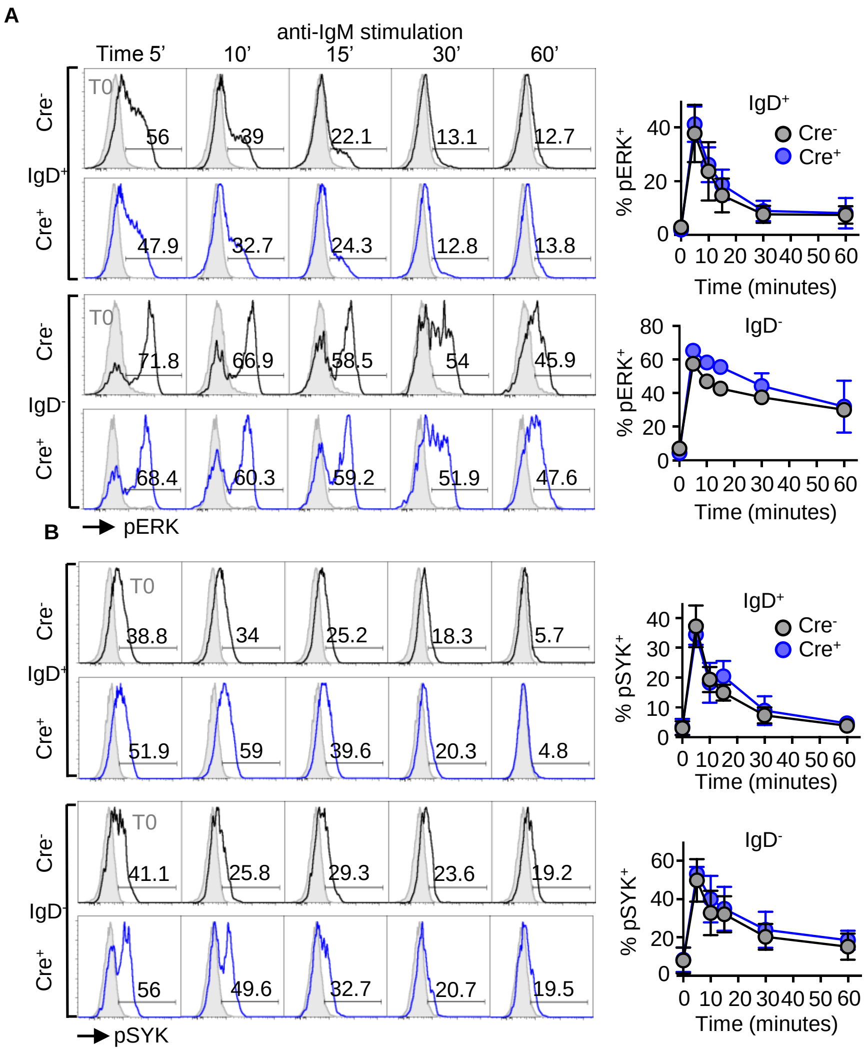

Figure S6. *Aip^fl/fl^* Cre⁺ mice have no differences in ERK or SYK phosphorylation following BCR stimulation; related to Figure 3

IgD⁻ B cells from *Aip^fl/fl^* Cre⁺ and Cre⁻ mice were stimulated with anti-IgM (10µg/ml) and examined for the expression of phosphorylated (**A**) ERK and (**B**) SYK from 5 to 60 minutes post-stimulation. Grey histograms show expression at time 0 (T0). Data expressed as percentage from time 0. Results are from two independent experiments with 1-2 mice per experimental group.
